# Determinants of Patients’ Adherence to Malaria Treatment in the Democratic Republic of the Congo

**DOI:** 10.3390/tropicalmed7070138

**Published:** 2022-07-18

**Authors:** Nsengi Y. Ntamabyaliro, Christian Burri, Yves N. Lula, Didier B. Nzolo, Aline B. Engo, Mireille A. Ngale, Hippolyte N. Situakibanza, Eric S. Mukomena, Gauthier K. Mesia, Samuel M. Mampunza, Gaston L. Tona

**Affiliations:** 1 Unité de Pharmacologie Clinique, Faculté de Médecine, Université de Kinshasa, Kinshasa P.O. Box 834, Democratic Republic of the Congo; yves.lula@unikin.ac.cd (Y.N.L.); didiernzolo@yahoo.fr (D.B.N.); aline.engo@unikin.ac.cd (A.B.E.); ngalemireille@gmail.com (M.A.N.); mesia.kahunu@unikin.ac.cd (G.K.M.); mampunza552@gmail.com (S.M.M.); tonalutete@gmail.com (G.L.T.); 2 Swiss Tropical and Public Health Institute, Kreuzstrasse 2, 4123 Allschwil, Switzerland; christian.burri@swisstph.ch; 3 Department of Pharmaceutical Sciences, University of Basel, Petersplatz 1, 4001 Basel, Switzerland; 4 Département de Médecine Tropicale, Faculté de Médecine, Université de Kinshasa, Kinshasa P.O. Box 834, Democratic Republic of the Congo; situakibanza.nani@unikin.ac.cd; 5 Département de Médecine Interne, Faculté de Médecine, Université de Kinshasa, Kinshasa P.O. Box 834, Democratic Republic of the Congo; 6 Programme National de Lutte Contre le Paludisme, Ministère de la Santé, Av. Du Tourisme Commune de Ngaliema, Kinshasa P.O. Box 3088, Democratic Republic of the Congo; mukomena3@gmail.com; 7 Faculté de Médecine, Université de Lubumbashi, Lubumbashi P.O. Box 1825, Democratic Republic of the Congo; 8 Centre Neuropsychopathologique, Faculté de Médecine, Université de Kinshasa, Kinshasa P.O. Box 834, Democratic Republic of the Congo

**Keywords:** malaria, treatment, adherence

## Abstract

(1) Background: Malaria heavily affects the Democratic Republic of the Congo (DRC) despite the use of effective drugs. Poor adherence to malaria treatment may contribute to this problem. (2) Methods: In one rural and one urban health area in each of the 11 former provinces of the DRC, all households with a case of malaria in the 15 days preceding the survey were selected and the patients or caregivers were interviewed. Adherence to malaria treatment was assessed by self-declaration about its completion. Logistic regression was used to assess predictors. (3) Results: 1732 households participated. Quinine was the most used drug; adherence to artesunate–amodiaquine was the lowest and the main reason for treatment discontinuation was adverse reactions. Predictors of adherence were residence in an urban area, university education, catholic religion, and adoption of recommended behaviour towards a malaria case. Adherence was significantly lower for responders who obtained information on antimalarials from Community Health Workers (CHW). (4) Conclusions: Usage of recommended drugs and adherence to malaria treatment need to be promoted, especially in rural areas, and CHW involvement needs to be improved. Awareness messages need to be made accessible and comprehensible to poorly educated populations and churches need to be involved.

## 1. Introduction

Malaria still heavily affects the Democratic Republic of the Congo (DRC), despite the use of powerful control tools, among which are effective and safe drugs.

The World Health Organization (WHO) and the National Malaria Control Program (Programme National de Lutte contre le Paludisme (PNLP)) recommend that each case of malaria be treated as quickly as possible, at latest within 24 h, and after parasitological confirmation, with effective drugs, in particular artemisinin-based combination therapies (ACTs) [1,2]. In the DRC, artemether–lumefantrine and artesunate–amodiaquine are recommended for the management of uncomplicated malaria. However, correctly prescribing the right antimalarial is not the end of the story. Patients must adhere to the treatment regimen to benefit from it, which, unfortunately, is not always the case [3,4].

As malaria is a transmissible disease with a uniquely human reservoir [5,6], the correct treatment of all cases is crucial for the individual, but also for disease control. Failure to adhere to antimalarial treatment increases transmission of the disease. Mathematical modelling has shown that the infectivity of an untreated patient is 29–51 times higher than that of a correctly treated patient [7]. However, adherence to the treatment of illness in general and to antimalarial treatment in particular is not always perfect [4,8,9,10,11,12,13]. Therapeutic adherence depends on several factors, which are not always easy to identify [14]. Nevertheless, it is possible to measure the degree of adherence, identify the most affected drugs, and identify some determinants. This will help to assess the real impact of antimalarial interventions related to case management and to propose more appropriate improvement measures. Studies conducted in Ethiopia [10] identified patient-related factors (fear of adverse effects and forgetfulness) and community-related factors such as the perception of drug efficacy. A study conducted in Ghana [8] identified other factors, including knowledge of the drug and its beneficial effects. The present study was designed to determine the adherence to malaria treatment in the DRC at a community level, and to contribute to the understanding of potential aberrations from the prescribed drug regimens.

## 2. Materials and Methods

This is part of a descriptive cross-sectional study that was conducted in urban and rural health areas in the DRC in order to assess determinants of the rational use of antimalarial in health facilities and at a community level, used the FURAP study (FURAP = Facteurs de l’Usage Rationnel des AntiPaludiques). Knowledge of the recommended antimalarials and health seeking behaviour of patients towards a suspected malaria case have been published in a previous article [15]. The current article addresses drugs actually used by patients, therapeutic adherence, and its determinants.

### 2.1. Study Setting

A study on rational use of antimalarials in health facilities, the URAP study (URAP = Usage Rationnel des AntiPaludiques) was carried out in 2014, which selected one general referral hospital (GRH), one rural health centre (RHC), and one urban health centre (UHC) in each of the former 11 provinces of the DRC [16]. The FURAP study, of which this article is a part, assessed the determinants of the rational use of antimalarials in the health facilities selected by the URAP study and in the catchment areas (health areas).

In the selected areas, all patients treated for malaria at the health centre in the two weeks preceding the survey were identified. The addresses of these patients were extracted and the listed households were visited by the study teams. The patient or caregiver were interviewed if they agreed to sign the informed consent. If the responder was a minor, the parent or legal guardian provided informed consent.

### 2.2. Data Collection

A questionnaire was developed and pre-tested in 20 households in the Mont Amba Health Zone in Kinshasa. After this validation, the questionnaire was administered by investigators to the patient or caregiver of a sick child. Data were collected in March and April 2018. The following parameters were assessed: household size, age, sex, religion, main source of information on antimalarials, and level of education of the responder. Knowledge of the recommended antimalarial was verified by the ability to name these drugs. If the responder was able to name at least one recommended antimalarial without adding any non-recommended medication, he/she was classified as knowing the recommended antimalarials. Behaviour was assessed by asking what is done in the case of suspicion of malaria: Consulting the community health worker, the healthcare provider at the health centre, or going to a health institution for a rapid diagnostic test for malaria (m-RDT) was considered as the recommended behaviour. Treatment adherence was assessed by patient declaration on whether or not they had completed the treatment. A responder who declared to completed the full course of treatment was considered as adherent.

Data were entered using Epi-info 7, CDC Atlanta, and then exported to Microsoft Excel 360 (Microsoft Corporation) and were analysed by Stata 14 software (StataCorp. 2015. Stata Statistical Software: Release 14. College Station, TX, USA: StataCorp LP.).

### 2.3. Statistical Analysis

Variables were described by number or frequency. When necessary, Chi-square tests were used to compare them. A *p*-value < 0.05 was considered for statistical significance. Logistic regression was used to assess the determinants of adherence to antimalarial treatment. Respondent’s age, sex, level of education, religion, household size, knowledge of antimalarials recommended by the PNLP, source of information on antimalarials, and the behaviour in case of suspicion of malaria were considered as explanatory variables. After the bivariate analyses, all of the variables with a statistical significance of 0.2 and less, as well as all those considered likely predictors, were included in the multivariate model. The final model was obtained by backward elimination.

### 2.4. Ethical Considerations

All of the participants provided written informed consent before participating in the survey. Approval was obtained from the Ethics Committee of the Congo Protestant University under the number CEUP 0048 before the start of the study. Confidentiality of the patients’ information was maintained and the current analysis did not include individual identifiers.

## 3. Results

Characteristics of the Households

Here, 1732 households participated in the study. Overall, adherence to any treatment was 73.1%. Table 1 shows that the proportion of adherent patients was higher in urban areas, among those with a higher level of education, and those who had adopted a behaviour recommended by the PNLP. When the main source of information about antimalarials was community health workers, the proportion of adherent responders was the lowest (49.6%).

Table 2 shows that the most used drug was quinine (37.8%), followed by artesunate–amodiaquine (26.0%), and 3.1% used injectable monotherapies of artemisinin derivatives.

Treatment adherence was the lowest for quinine (61.3%), as shown in Table 3. Adherence to ASAQ and AL was 80% and 91.6%, respectively. Only 10 persons took other ACTs (artenimol–piperaquine and artemisinin-piperaquine) and all adhered to the treatment.

Table 4 shows that adverse reactions were the reason for the discontinuation of treatment in 64.5%, 56%, and 41.7%, respectively, for ASAQ, quinine, and AL. Quinine (7–10 days of treatment) is the only drug for which the length of the treatment and the difficulty of the dosing were mentioned as reasons for treatment interruption.

In the multivariate regression analysis shown in Table 5, residence, education, religion, behaviour, and the main source of information on antimalarials were independent predictors of adherence to antimalarial treatment. Responders living in urban area were more likely to adhere to malaria treatment compared with those living in rural areas (aOR = 1.6 [1.3–26], *p* = 0.000), university educated were more likely to adhere than illiterates (aOR = 1.7 [1.1–9.6], *p* = 0.040), and Catholics were more likely to adhere than Protestants (aOR = 1.5 [1.0–2.3] *p* = 0.043). Responders who adopted recommended behaviour towards a suspected malaria case were more likely to adhere than those with non-recommenced attitudes (aOR = 1.7 [1.2–2.4], *p* = 0.004). However, the likelihood of adhering to treatment decreased by 60% (aOR = 0.4 [0.3–0.7], *p* = 0.000) for the responders whose main source of information on antimalarials was community health workers compared with those for whom the main source was the medical staff.

## 4. Discussion

Malaria is a curable and preventable infectious disease; however, it continues to cause hundreds of thousands of deaths each year in the world, 13.2% of which are (about 82,700) in the DRC [17]. In order to coordinate the fight against this deadly disease in the DRC, a national program was set up, which has developed guidelines to enable the use of the most effective and safe tools to fight malaria. This is why artemisinin-based combination therapies are recommended as first-line treatment [18,19]. This study did not assess how the responders acquired malaria treatment. However, most of the time, out-of-pocket expenses were required, despite the availability of some of the drugs for free in public health facilities. This is because of the requested payments for medical consultation and lab exams before obtaining the drug for free; the poorest of the population prefer to spend the little money they have to buy medicines instead, and thus resort to self-medication [20]. In addition, in contrast with the recommendations, the most frequently used drug remains quinine, which is not provided for free. This is why poverty (inability to purchase full course of treatment) is one of the reasons for non-adherence in all drugs. Nevertheless, the most effective treatment will only be of any use if the patient takes it as recommended, in other words, if they adhere to it. This aspect is of paramount importance for a disease treated on an outpatient basis, such as uncomplicated malaria. Adherence can be measured by several methods. The reported study used self-reporting of the patient on whether or not they completed the full course of treatment.

In a previous study to compare adherence to AL, assessed by patient self-reporting or smart blister cards that recorded the date and time each pill was removed from the package, [21] Katia Bruxvoort et al. found that the two methods gave comparable results with regard to the completion of treatment (64% and 67%, respectively). A Senegalese study conducted by Souares et al. [22] found a correspondence of 72 to 90% between the declaration of adherence to the treatment and the plasma concentration of the drug. These studies were conducted in sub-Saharan countries (Tanzania and Senegal), with comparable cultural socio-economic situations to the DRC. Patient declaration is therefore a comparably simple, but reliable method for estimating treatment adherence.

Adherence to injectable monotherapies of artemisinin derivatives was 100%; these drugs were likely administered by health personnel, thus increasing compliance. Adherence to quinine was the lowest. Quinine has been used for over 400 years [23]; it was the first chemical compound that was successfully used to treat an infectious disease [24] and has undisputedly saved many lives. However, quinine is not recommended as first-line therapy by the PNLP because of its narrow therapeutic window, worse average treatment outcome compared with modern drugs, and complex application, but it still was the most frequently used drug for the management of uncomplicated malaria. In the DRC, the popularity of quinine is such that the word “quinine” is used in some local languages to designate any tablet. Hence, health staff, caregivers, and patients have grown up in a culture of positive perception of quinine and often stick to this compound as the treatment of choice against all contradicting facts. More than half of the patients discontinued treatment prematurely, mainly because of adverse drug reactions, which are favoured by the low therapeutic index [24] and the complexity of the therapeutic regimens [24]. Numerous studies have also shown the significant superiority of ACTs over quinine in terms of a lower case fatality rate [25,26,27], as well as a reduction in gametocytes [28,29], the transmissible form of malaria. For all these reasons, it is of great importance to work towards behaviour change in communities and health care staff towards using more recommended drugs.

Poor adherence to ASAQ is another important and controversial issue. Certain studies have shown a higher proportion of adverse events with ASAQ compared with other antimalarials [30,31], while others have shown a similarity in terms of tolerance [32]. For our study, the main reason for non-adherence to ASAQ was the occurrence of adverse reactions. This resulted in a higher proportion of patients who discontinued ASAQ treatment (64.5%) compared with AL (41.7%) and quinine (56.4%). This phenomenon had already been observed by Likwela et al. [33], who identified adverse reactions as the main factor for non-adherence to ASAQ treatment. Lula et al. found adverse events in ASAQ to be higher than in AL and quinine–clindamycin [34]. A study performed in Sierra Leone also noted a lower adherence to ASAQ compared with AL and a higher proportion of adverse reactions [35]. This lower adherence to ASAQ was assessed as the number of tablets taken, whereas adherence to correct times of intake of AL was lower [35]. Indeed, AL has a more complex medication regimen with two doses per day, of which the first two are spaced by 8 h and all of the following doses are spaced by 12 h, making it more difficult to comply with, whereas ASAQ is taken in a single daily dose. Taking this into account, one should be more cautious in the assertion of better adherence to AL; our study did not consider adherence to the time of drug intake. In this context, it is worth mentioning that it is easier for the patient to remember whether they have completed their treatment or not, than to remember the exact time they took each pill.

### Determinants of Adherence to Malaria Treatment

The threat that malaria poses to the community makes it necessary to study not only the practices, but also the determinants, in order to propose appropriate corrective measures. In our study, urban residence, university education, catholic religion, and the adoption of a recommended behaviour towards a malaria case were independent determinants of adherence to antimalarial treatment. Of note, community health workers as a source of information on the recommended antimalarial drugs was an independent determinant of non-adherence. Indeed, the likelihood of adhering to antimalarial treatment decreased by 60% among responders for whom the main source of information on antimalarials was the CHWs (aOR = 0.4 [0.2–0.6], *p* = 0.000). This finding prompts reflection on the role and quality of care of community health workers in the fight against malaria. This is of high relevance, as the WHO have stated that CHWs are of paramount importance in the early detection and correct treatment of cases [36], and they have been considered to be the backbone of malaria elimination activities [37]. While the CHW’s intervention has a negative impact on the therapeutic adherence of patients, it also failed to be a determinant of knowledge and recommended behaviour in our previous study [15]. This raises questions about the reasons for the deficient performance of these key stakeholders in malaria diagnosis and treatment, and asks for an in-depth follow up on the root causes followed by targeted training. Delacotte et al. showed their importance in the fight against malaria in the DRC, but had already foreseen the difficulty of maintaining their performance over time [38].

CHWs are the closest health worker to the population. They reach them where they eat, work, or worship [39]. This could constitute an advantage in health communication, but could also constitute a disadvantage if the CHW does not have enough arguments to explain the reasons for the choices made by the PNLP regarding malaria treatment. CHWs often come from economically disadvantaged populations [40] and often work voluntarily [39,40], which may negatively impact performance in health communication. Their work should be recognized, valued, and properly remunerated, as suggested by the WHO and the United Nations Children’s Fund (UNICEF), who spoke about the institutionalization of community care [41] for more efficient performance. To be effective, CHWs will need to be properly trained, integrated into the health system, and properly motivated; otherwise, their intervention may be counterproductive.

Urban residence is one of the independent determinants of adherence to antimalarial treatment. This could be related to the higher economic level of the urban population. Indeed, many studies have shown a relationship between low socioeconomic level and non-adherence to treatment, including antimalarial treatment [12,14,35]. This also constitutes a call to pay particular attention to the inhabitants of rural areas in the management of malaria in the DRC.

Another determinant is education. University-level education is an independent determinant of treatment adherence; this seems obvious given the better ability to understand the treatment and all its implications for patients. An Ethiopian study identified illiteracy and lack of knowledge of drugs among the determinants of non-adherence (33). This led to the concept of health literacy, first introduced in Canada by Rootman and Gordon-El-Bihbety, who defined it as the ability to access, understand, evaluate, and communicate information in ways that promote, maintain, and improve one’s health in a variety of settings over the course of life [42]. Health literacy is linked to the level of education [43] and is an important determinant of adherence to treatment [8,12,44,45]. Improving health literacy involves improving the level of education of the population and involves sectors other than health. This underlines the necessarily multifactorial nature of the fight against malaria.

Patients who have adopted the recommended behaviour in the case of suspected malaria are also more likely to adhere to treatment. First, they adopt the recommended behaviour in case of suspicion of malaria, as was shown in our previous study [15], and then they adhere to the treatment they have been prescribed. This suggests that the messages likely to help the population to know and follow the recommendations on the management of malaria will also improve adherence to all measures, including malaria treatment. Messages on correct behaviour must therefore be strengthened and increasingly adapted to populations.

Our results suggest that religion is a significant element in the fight against malaria as well. Indeed, belonging to the catholic religion was a determinant of adherence to antimalarial treatment. This is in line with a Nigerian study that showed the catholic religion to be a determinant of a better attitude towards health matters [46]. Adherence to antimalarial treatment was lower among the followers of evangelical churches; however, multivariate logistic regression did not confirm this variable to be a determinant of non-adherence. It could be argued that evangelical churches preach miracle healing more than the catholic church, and that this might influence adherence to treatment. The fact that atheists (who therefore do not believe in miracle healing at all) have the best adherence to treatment (83.3% versus 74.3% for Catholics and 64.4% for evangelical Christians) seems to reinforce this idea, but the sample size was low (six atheists in our sample). In any case, these results underline the importance of religion in the field of health for the Congolese population who are known to be deeply religious [47]. This influence indicates that spreading the messages on the fight against malaria in the churches could be a good idea. The involvement of religious leaders in the fight against malaria would be a strategy with the potential to bring positive results.

## 5. Conclusions

The proven efficacy of malaria drugs fails to produce the expected positive results in the real world in the DRC. One of the causes identified is the non-adherence of patients to the treatment schedules recommended by the PNLP. The most widely used drug in the DRC, despite its many disadvantages, is still quinine, which is not a recommended first-line treatment and is also suboptimal in terms of non-adherence, mainly because of adverse drug reactions. Residence in an urban environment, a university education, belonging to the catholic religion, and the ability to adopt the behaviour recommended by the PNLP are determinants of adherence to antimalarial treatment. CHWs, on the other hand, seem to have a negative influence on adherence to antimalarial treatment. The motivation and better capacity of community health workers may contribute to improving the results of the fight against malaria in the DRC. These elements, taken together, ask for an improvement in the health system in the DRC, particularly in rural areas, to adapt the awareness messages and make them more accessible to people with a poor education, and to include churches in the fight against malaria.

## Figures and Tables

**Table 1:** Characteristic of the responders and adherence to malaria treatment.

	Adherence to Any Antimalarial Treatment		*p*-Value
	*N*	Percentage	
Residence of the responder (*n* = 1184)			
Rural area (631)	422	66.8%	0.000
Urban area (553)	423	76.5%	
Education of the responder (*n* = 1294)			
Illiterate (*n* = 66)	45	68.2%	0.018
Primary school (*n* = 233)	153	65.7%	
Secondary school (*n* = 760)	527	69.3%	
University (*n* = 235)	184	78.3%	
Age of the responders (years) (*n* = 1278)			
≤18 (*n* = 13)	9	69.2%	0.693
18–50 (*n* = 981)	684	69.7%	
50–65 (*n* = 221)	151	68.3%	
≥65 (*n* = 63)	48	76.2%	
Sex of the responders (*n* = 1290)			
F (*n* = 711)	502	70.6%	0.510
M (*n* = 579)	399	68.9%	
Size of the household (*n* = 1295)			
<6 persons (*n* = 473)	342	72.3%	0.203
6–10 persons (*n* = 625)	434	69.4%	
˃10 persons (*n* = 197)	129	65.5%	
Informed about antimalarials (*n* = 1312)			
No (*n* = 441)	311	70.5%	0.724
Yes (*n* = 871)	606	69.6%	
Religion of the responder (*n* = 1270)			
Protestant (*n* = 425)	298	70.1%	0.187
Catholic (*n* = 421)	313	74.3%	
Evangelical (*n* = 303)	195	64.4%	
Kimbanguist (*n* = 34)	22	64.7%	
Muslim (*n* = 73)	53	72.6%	
African religions (*n* = 7)	5	71.4%	
Atheist (*n* = 6)	5	83.3%	
Know antimalarials (*n* = 875)			
No (*n* = 247)	168	68.0%	0.408
Yes (*n* = 628)	445	70.9%	
Main source of information on antimalarials (*n* = 871)			
Medical staff (*n* = 395)	289	73.2%	0.000
Media (*n* = 266)	191	71.8%	
Community Health Worker (*n* = 115)	57	49.6%	
Relatives (*n* = 67)	52	77.6%	
Pharmacy (*n* = 1)	1	100.0%	
Other (*n* = 24)	16	66.7%	
Training (*n* = 3)	3	100.0%	
Behaviour in case of suspected malaria (*n* = 1295)			
Non-recommended (*n* = 816)	537	65.8%	0.000
Recommended (*n* = 479)	370	77.2%	

**Table 2:** Drugs used by the responders (*N* = 1605).

Antimalarial	Number (%)
Quinine	606 (37.8%)
ASAQ	418 (26.0%)
AL	159 (9.9%)
Artemisinin derivatives used as monotherapy: artemether inj. (*n* = 40), α-β-arteether (*n* = 5), artesunate inj. (*n* = 4);	49 (3.1%)
Other ACTs: artenimol–piperaquine (*n* = 9); artemisinin–piperaquine (*n* = 1)	10 (0.6%)
Other antimalarials (SP, chloroquine)	56 (3.5%)
Herbal medicines	47 (2.9%)
Non antimalarials	282 (17.6%)

ASAQ = artesunate/amodiaquine; AL = artemether/lumefantrine; SP = sulfadoxine-pyrimethamine; ACT = artemisinin-based combination therapy.

**Table 3:** Adherence to different malaria treatments.

Antimalarial (*n*)	Adherence Number (%)
Quinine (*n* = 573)	351 (61.3%)
ASAQ (*n* = 395)	316 (80.0%)
AL (*n* = 155)	142 (91.6)
Other antimalarials (SP, chloroquine) (*n* = 56)	55 (98.2)
Artemisinin derivatives monotherapies (*n* = 43)	43 (100%)
Other ACTs (*n* = 10)	10 (100%)
Herbal medicines (*n* = 38)	33 (86.8%)

ASAQ = artesunate/amodiaquine; AL = artemether/lumefantrine; SP = sulfadoxine–pyrimethamine; ACT = artemisinin-based combination therapy.

**Table 4:** Reason for treatment discontinuation (non-adherence).

Reason for Discontinuation	Number (%)
QUININE	
Adverse reaction	114 (56.4%)
Inability to purchase full course of treatment	25 (12.4%)
Resolution of symptoms	23 (11.4%)
Drug considered ineffective	16 (7.9%)
Other	10 (5.0%)
Too long a course of treatment	8 (4.0%)
Dosage difficult	6 (3.0%)
ARTESUNATE–AMODIAQUINE	
Adverse reaction	49 (64.5%)
Other	8 (10.5%)
Inability to purchase full course of treatment	8 (10.5%)
Drug considered ineffective	6 (7.9%)
Resolution of symptoms	5 (6.6%)
ARTEMETHER–LUMEFANTRINE	
Adverse reaction	5 (41.7%)
Other	4 (33.3%)
Resolution of symptoms	1 (8.3%)
Drug considered ineffective	1 (8.3%)
Inability to purchase full course of treatment	1 (8.3%)

**Table 5:** Determinants of treatment adherence.

	Bivariate Regression			Multivariate Regression		
	OR	IC 95%	*p*-Value	aOR	IC 95%	*p*-Value
Residence of the responder						
Rural	1			1		
Urban	1.6	1.2–2.1	0.000	1.8	1.3–2.6	0.001
Education of the responder						
Illiterate	1			1		
Primary school	0.9	0.5–1.6	0.735	1.6	0.6–4.8	0.382
Secondary school	1.1	0.6–1.8	0.820	2.0	0.7–5.5	0.195
University	1.7	0.9–3.1	0.091	3.2	1.1–9.6	0.040
Religion of the responder						
Protestant	1			1		
Catholic	1.2	0.9–1.7	0.169	1.5	1.0–2.3	0.043
Evangelical	0.8	0.6–1.1	0.104	1.2	0.7–1.8	0.510
Kimbanguist	0.8	0.4–1.6	0.496	1.0	0.4–2.5	0.974
Muslim	1.1	0.6–2.0	0.688	1.9	0.9–3.9	0.90
African religion	1.1	0.2–5.5	0.948	1.0	0.1–17.4	0.989
Atheist	2.1	0.2–18.3	0.496	2.3	0.2–24.2	0.498
Behaviour in case of suspected malaria						
Non-recommended	1			1		
Recommended	1.8	1.4–2.3	0.000	1.7	1.2–2.4	0.004
Main source of information on antimalarials						
Medical staff	1			1		
Media	0.9	0.7–1.3	0.701	1.0	0.6–1.4	0.850
CHW	0.4	0.2–0.6	0.000	0.4	0.3–0.7	0.000
Relatives	1.3	0.7–2.4	0.445	1.4	0.7–2.9	0.317
Other (church, social media, etc.)	0.7	0.3–1.8	0.489	0.7	0.3–1.9	0.507

## Data Availability

All excel files are available from the Figshare database doi:10.6084/m9.figshare.19704421.
